# Wanting to Forget: Intrusive and Delusional Memories from Critical Illness

**DOI:** 10.1155/2020/7324185

**Published:** 2020-03-24

**Authors:** Lauren Doig, Kevin Solverson

**Affiliations:** Department of Critical Care Medicine, Cumming School of Medicine, University of Calgary, Calgary, Alberta, Canada

## Abstract

*Introduction*. Delusional and fearful memories after critical illness are observed in up to 70% of patients post critical illness. However, they often go unrecognized after patients leave the intensive care unit (ICU). *Case Presentation*. A 40-year-old male was admitted to the ICU with community-acquired pneumonia and multiorgan failure requiring mechanical ventilation and renal replacement therapy. He developed protracted delirium and severe ICU-acquired weakness but was eventually discharged home. The patient returned to a follow-up clinic two months post-ICU discharge and revealed that he was suffering anxiety from memories in the ICU of different staff trying to harm and kill him, including being repeatedly suffocated. By providing context to the memories, the patient had significant relief in his anxiety. *Conclusions*. Intrusive memories contribute to psychological morbidity post critical illness, including posttraumatic stress disorder (PTSD) and reduced health-related quality of life. The majority of critical illness survivors do not share their intrusive or frightening memories, and therefore, most healthcare professionals are unaware of the problems they can pose. Assessment of patients' memories from the ICU is essential and may create the opportunity to help patients place memories into context and improve psychological morbidities.

## 1. Introduction

The mortality from critical illness is decreasing, but many patients develop multiple new and long-lasting morbidities related to critical illness. The term postintensive care syndrome (PICS) describes the physical, cognitive, and mental health comorbidities that commonly arise post critical illness [1]. Physical comorbidities that cause a reduced health-related quality of life (HRQL) as a sequela of critical illness are perhaps easy to understand because the sequelae may be readily visible and require ongoing clinical management. Perhaps as many patients also report impairments to their mental health, including depression, anxiety, and posttraumatic stress disorder (PTSD), all of which may be more difficult to recognize. An important contributor to mental health that commonly goes unrecognized is the memories patients recall from their ICU stay.

## 2. Case Presentation

The patient described in this report provided written consent prior to publication. A previously healthy 40-year-old Caucasian male was admitted to ICU with community-acquired pneumonia. His course was complicated by severe hypoxic respiratory failure and multiorgan dysfunction, including shock, hepatic dysfunction, delirium, and acute renal failure necessitating renal replacement therapy. He had a protracted stay due to respiratory and muscle weakness associated with critical illness myopathy confirmed by electromyography. His ICU stay was complicated by episodes of delirium necessitating the use of anxiolytics and neuroleptic medications. He was seen at an ICU follow-up visit 2 months postdischarge. Response to structured questionnaires identified significant concerns with anxiety, depression, and sleep disruption. During the clinical meeting, the patient confided of a persistent memory that one of the team members was a witch, mixing potions to poison him. His wife provided a collateral history that, although the patient was normally mild, soft-spoken, and gentle, he was singularly curt and rude to this staff member without explanation. He also had memories of a “German” man in a white “suit” that when close to him in the ICU made him feel short of breath, and the patient described subsequent disorganized frightening/disquieting dreams of the same individual. The patient was able to provide specific and explicit descriptions that permitted both individuals to be identified. He also had persistent vivid memories of forcible confinement in a cave, where he could not escape, apart from a series of white doors, none of which seemed safe or from which he could exit. The patient was provided descriptions of the staff (one a nurse with longer grey hair, the other an RT known for always wearing a buttoned white lab coat) and descriptions of their roles with possible explanations for the “memories” (the nurse mixing/administering intravenous medications; the RT suctioning the patient with associated sense of dyspnea during periods of being disconnected from the ventilator) which provided some emotional relief to the patient. The memories of specific staff slowly faded, but the vivid disquieting dreams of confinement persisted for years.

## 3. Discussion

The reported prevalence of delusional and hallucinatory memories from critical illness ranges from 25 to 73% [2, 3]. It is common for patients who have delusional memories also to recall factual events, but a small percentage of patients will solely recall delusional events [2]. In the critical care population, delusional memories are frequently frightening and contain persecutory themes. In this case description, the patient thought an ICU staff member was repeatedly trying to hurt him, a common presecretory theme from the ICU [4]. Wade et al. [4] describe a variety of delusional themes from patients with traumatizing memories after critical illness including purposely being poisoned, assaulted, tortured, or staff conspiring against them. The intrusive delusions and hallucinations are long-lasting and remain vivid for months after critical illness [4, 5].

ICU-acquired delirium occurs in up to 80% of the patients severely ill in the ICU [6] and is thought to be a significant risk factor for the development of delusional memories after critical illness. The presence of delirium has been associated with patients recalling fewer factual memories, and an increase in intrusive delusional memories [7]. Sedative and analgesic medications commonly used in the ICU include benzodiazepines and opioids, and they are hypothesized to have an effect on memory recall in the ICU [8]. However, a recent post hoc analysis of a randomized controlled trial of sedation protocols (light sedation versus usual care) found no difference in memory recall or the presence of delusional memories between the groups [5].

The prevalence of PTSD after critical illness ranges from 8 to 51%, and intrusive memories may be an important risk factor for its development [1]. Patients who experienced nightmares and recalled stressful memories two months after the ICU had a higher prevalence of PTSD, anxiety, and depression [3]. More than 50% of patients with PTSD six months after critical illness describe memories of panic and suffocation while in the ICU [9]. Jones et al. [10] found that patients with no factual recall of the ICU but with the presence of delusional memories scored higher on anxiety, depression, and PTSD scales in comparison to patients with factual events recalled. Wade et al. [4] found that patients with PTSD after critical illness are more likely to have intrusive memories secondary to delusions than those with intrusive memories from real or factual events.

Limited data is available on interventions to improve psychological morbidity and memories after critical illness. Jones et al. [11] studied the use of ICU diaries, documenting the patients' ICU course with written descriptions and pictures, and examined its effect on PTSD and post-ICU memories. The incidence of new PTSD was significantly reduced (5% treatment vs. 12% control) in the diary group. The authors hypothesize that the use of diaries fills in the patient's memory and may give context to delusional memories and prevent psychological distress and morbidity. As described in our case report, simply giving the patient context of their delusional memory, such as the process of endotracheal suctioning, the unpleasant symptoms it can evoke, and that no one was trying to harm him, helped the patient realize his memories were unlikely to be factual and reduced his anxiety.

The majority of patients with psychological distress related to ICU memories want to discuss these issues with a healthcare professional [4]. However, at six months after critical illness, less than 20% of patients have discussed their admission to the ICU with a primary care physician [12]. Specialized ICU follow-up clinics after critical illness are not the standard of care in most countries, leaving much of the burden of recognizing and caring for post-ICU comorbidities up to the primary health care provider. Therefore, it is crucial to increase healthcare providers' awareness of post-ICU morbidity, including memories from the ICU and the associated mental health consequences they may have so that appropriate interventions may be provided.
